# The urinary microbiome in patients with refractory urge incontinence and recurrent urinary tract infection

**DOI:** 10.1007/s00192-018-3679-2

**Published:** 2018-06-26

**Authors:** Zhuoran Chen, Minh-Duy Phan, Lucy J. Bates, Kate M. Peters, Chinmoy Mukerjee, Kate H. Moore, Mark A. Schembri

**Affiliations:** 1 Department of Urogynaecology, St George Hospital, University of New South Wales, Kogarah, NSW 2217 Australia; 2 School of Chemistry and Molecular Biosciences, The University of Queensland, Brisbane, Queensland 4072 Australia; 3 Australian Infectious Diseases Research Centre, The University of Queensland, Brisbane, Queensland 4072 Australia; 4 Division of Microbiology, SEALS, St George Hospital, Kogarah, New South Wales Australia

**Keywords:** Urinary urge incontinence, Refractory detrusor overactivity, Urinary tract infection, Escherichia coli, Urinary microbiome, fimH

## Abstract

**Introduction and hypothesis:**

Urinary urge incontinence is a chronic, debilitating condition that is difficult to treat. Patients refractory to standard antimuscarinic therapy often experience recurrent urinary tract infections (rUTIs). The microbiota of these refractory patients with rUTI remains unexplored.

**Methods:**

A midstream urine (MSU) sample was collected from patients with refractory urge incontinence and coexistent rUTI during acute symptomatic episodes. Culture-based diagnosis was performed using routine microbiological methods. Culture-independent profiling was performed using bacterial 16S RNA profiling. *E. coli* strain typing was performed by amplicon pyrosequencing of the *fimH* gene.

**Results:**

Over 2 years, 39 patients with refractory urge incontinence and coexistent rUTI were studied, yielding 9 severely affected cases. These 9 patients were carefully monitored for a further 2 years, resulting in the collection of 102 MSU samples, 70 of which were diagnosed as UTI (median of 8 UTIs/woman). Culture-independent analysis of 38 of these samples revealed the existence of a diverse urinary microbiota. Strain typing of *E. coli* identified instances of rUTI caused by the same persisting strain and by new infecting strains.

**Conclusions:**

Patients with refractory urge incontinence and coexistent rUTI possess a diverse urinary microbiota, suggesting that persistent bladder colonisation might augment the pathology of their chronic condition.

## Introduction

Urge incontinence is a debilitating clinical condition characterised by urinary frequency, urgency and nocturia [1]. Patients experience bladder detrusor muscle spasms that can cause overwhelming leakage, making this a health condition that dramatically decreases the quality of life in approximately 17% of women over the age of 40. Clinical diagnosis is based on a standard urodynamic test that observes abnormal detrusor muscle contractility, termed detrusor overactivity (DO). Patients non-responsive to antimuscarinic therapy are described as “refractory”.

Recent studies employing culture-independent sequencing have revealed that the bladder of patients with urge incontinence and refractory DO is not sterile [2]. These patients harbour a diverse urinary microbiome [3–5], indicating the previously unrecognised role of bacterial consortia in the pathophysiology of their disease. In line with these observations, others have reported that 40–60% of patients with refractory DO suffer recurrent urinary tract infections (rUTIs) [6], which may also be involved in the pathogenesis of their condition [7]. The existence of intracellular bacteria in exfoliated urothelial cells from patients with urge incontinence has also been demonstrated, providing a link to a bladder reservoir for rUTI [8]. The rising incidence of UTIs caused by antibiotic-resistant bacteria represents a problem of increasing concern that complicates the management of refractory DO. As a result, patients with refractory DO who suffer from rUTI have become an increasing burden upon health care systems as they seek help for a disabling problem that has no effective cure.

The discovery of a urinary microbiome in women with urge incontinence provides a new opportunity to understand this poorly characterised chronic condition. The few studies reported in this area to date have primarily compared the diversity of the urinary microbiome in patients with urge incontinence against control subjects [3–5]. However, there are no studies that have examined the urinary microbiome in patients with refractory DO and rUTI. Here we applied culture-independent sequencing in combination with standard diagnostic microbiology to investigate changes in the urine microbiota of women with urodynamically proven refractory DO and concomitant UTI over a 2-year time period. We show that these women exhibit diversity in their urinary microbiome over time, and provide evidence to support infection with both new and pre-existing organisms in the development of recurrent, symptomatic UTI.

## Materials and methods

### Study protocol

Study participants were recruited from a regional urogynaecology centre in a major public hospital. Ethics approval was obtained from the local hospital Research Ethics Committee (LNR/14/POWH/345). Written consent was obtained from all participants.

Patients were recruited using the following criteria:

1. A history of rUTIs, defined as ≥2 culture positive infections in 6 months or ≥ 3 infections in 1 year, as previously described [9]
2. No anatomical causes of rUTIs, as shown by cystoscopy
3. Proven DO via urodynamic testing along with a clinical diagnosis of refractory DO (defined as failure to respond to conservative therapy [bladder training and lifestyle changes, e.g. reduced caffeine intake] and > 2 antimuscarinic agents for more than 12 months)

Case notes were obtained for all patients to assess their history of rUTI over a period of 24 months; this included previous prolapse or incontinence surgery and information obtained through consultation with local general practitioners (Table 1). All postmenopausal women received topical per vaginal oestriol 1 mg/g cream 3 times weekly. Voiding dysfunction was defined as residual urine volume of >100 mL, which did not require self-catheterization. Thirty-nine consecutive women who met these criteria were observed over a 24-month period. Patients who had persistent or recurrent bacterial cystitis or classical cystitis were chosen for 16 s rRNA analysis.

**Table 1:** Patient demographic data

Patient number	Age	Type of incontinence	Comorbidities	Previous gynaecological surgery	Number of UTIs during the preceding 24 months	Number of UTIs during the 24 months of the study	Percentage* E. coli* during the 24 months of the study	Organisms isolated during the 24 months of the study	Antibiotic resistance
1	73	Refractory DO + stress incontinence	Obesity	Prolapse surgery + incontinence surgery	9	15 proven UTIs, 1 mixed growth	9/15 (60%)	*E. coli*,* E. coli* (ESBL),* E. faecalis*,* K. oxytoca*,* K. pneumoniae*	AMX, TMP, LEX
2	75	Refractory DO	Obesity, OSA	Prolapse surgery + incontinence surgery	16	17 proven UTIs, 2 mixed growths	5/17 (29.4%)	*E. coli*,* C. freundii*,* E. faecalis*,* Klebsiella* spp.	AMX, NIT, TMP, LEX
3	78	Refractory DO	Hypertension, hypothyroidism	Prolapse surgery + incontinence surgery	3	2 proven UTIs, 8 mixed growths	1/2 (50%)	*E. coli*, group B* Streptococcus*	AMC, TMP
4	57	Refractory DO	Obesity, previous gastric band, OSA, T2DM	Incontinence surgery	3	3 proven UTIs, 5 mixed growths	1/3 (33%)	*E.coli*,* P. mirabilis*,* Streptococcus* spp.	AMX, NIT
5	73	Refractory DO + stress incontinence	Cerebral vascular accident, primary hyperparathyroidism	Prolapse surgery + incontinence surgery	3	12 proven UTIs, 4 mixed growths	9/12 (75%)	*E. coli*,* E. faecalis*,* P. mirabilis*	AMX, TMP, NIT
6	80	Refractory DO + voiding dysfunction	Obesity, hypertension	Prolapse surgery + incontinence surgery	3	6 proven UTIs, 3 mixed growths	2/6 (33%)	*E. coli*,* E. faecalis*,* P. mirabillis*,* M. morganii*,* Streptococcus* spp.	AMX, AMC, LEX, TMP
7	79	Refractory DO + voiding dysfunction	T2DM, hypertension, obesity	Prolapse surgery	3	3 proven UTIs, 2 mixed growths	3/3 (100%)	*E. coli*	AMX
8	81	Refractory DO + stress incontinence	First-degree heart block	Incontinence surgery	4	7 proven UTIs, 1 mixed growth	4/7 (57%)	*E. coli*,* E. coli* (ESBL),* K. pneumoniae*	AMX, LEX, TMP, NIT, NOR
9	64	Refractory DO + mild stress incontinence	Nil	Incontinence surgery	3	5 proven UTIs, 1 mixed growth	5/5 (100%)	*E. coli*	TMP

*UTI* urinary tract infection, *DO* detrusor overactivity, *OSA* obstructive sleep apnoea, *T2DM* type 2 diabetes mellitus,* ESBL* extended-spectrum b-lactamase, *AMX* amoxicillin, *AMC* amoxicillin-clavulanic acid, *LEX* cephalexin, *NIT* nitrofurantoin, *NOR* norfloxacin, *TMP* trimethoprim

### Sample collection and microbiology

Urine collection was performed when patients experienced worsening of lower urinary tract symptoms (LUTS) such as urgency, frequency, or urge incontinence; however, dysuria and foul-smelling urine were not required for entry to the study. Patients provided midstream urine (MSU) specimens with careful labial toilet by parting the labia and rinsing the peri-urethral area with water [8]. Half of each MSU sample was sent to a single microbiology unit, where culturing and diagnosis was performed using a threshold of >10^3^ colony-forming units (CFU)/mL to identify the dominant infecting organism/s [8]. Antimicrobial susceptibility profiles were determined as described in the Calibrated Dichotomous Sensitivity Test manual [10]. The other half of the sample was immediately placed at 4°C, and within 30 min was stored at −20°C for subsequent bacterial DNA extraction and 16 s rRNA amplicon sequencing.

**Fig. 1:**
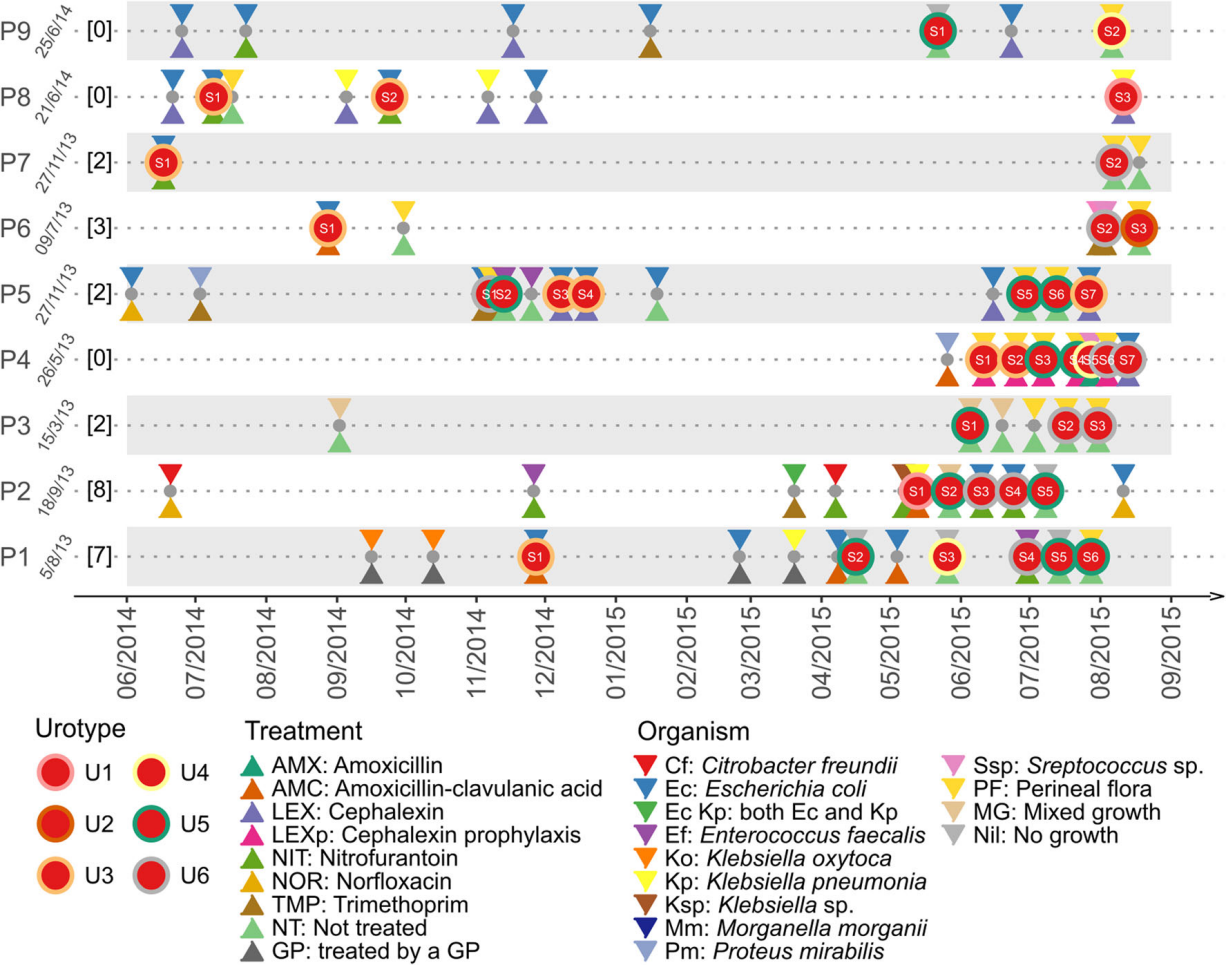
Overview of urine samples collected from each patient from June 2014 to September 2015. For each patient (P1 to P9), the date of recruitment to this study was indicated, followed (in* square brackets*) by the number of urine samples collected from the date of recruitment to June 2014.* Grey dots* indicate urine samples subjected to traditional microbiology culture only, whereas* red dots* indicate samples that were also subjected to 16S rRNA diversity profiling. Treatments at the time of collection were shown by a* triangle under the dots*, whereas organisms identified by traditional microbiology culture were indicated by an* inversed triangle above the dots*. Urotypes (more details in Fig. 2) were shown by* coloured rings outside the red dots*

Clinical treatment criteria for acute cystitis included symptoms of dysuria, suprapubic pain, and/or foul-smelling urine, in combination with a single organism reported on routine microbiology at >10^3^ CFU/mL (low count threshold) and pyuria (white cell count [WCC] >10 per high power field [HPF]). In the absence of dysuria, suprapubic pain, and/or foul-smelling urine, the microbiological criteria for treatment of acute UTI was >10^5^ CFU/mL with pyuria (WCC >100 per HPF). After acute UTI treatment, a post-therapy MSU was checked to be negative. Some patients received treatment in the community by their general practitioner, with less stringent criteria (Fig. 1). Where bacterial growth was >10^3^ CFU/mL, but no single dominant organism was cultured, these samples were reported as “mixed growth” and the patient was not treated. Bacterial isolates recovered from culture-positive MSU samples were stored in 20% glycerol at −80°C.

### 16S rRNA community profiling

Variable region 4 of the bacterial 16S rRNA gene was amplified directly from MSU samples using universal primers 515F and 806R. Preparation of the16S library was performed using the workflow outlined by Illumina (#15044223 Rev.B). Purified DNA was indexed with unique 8 bp barcodes using the Illumina Nextera XT 384 sample Index Kit A-D (Illumina FC-131-1002). Indexed amplicons were pooled in equimolar concentrations and sequenced (MiSeq, Illumina) using paired ends with V3 300 bp chemistry. The program mothur (version 1.37.3) [11] was used for 16S rRNA data analysis (www.mothur.org/wiki/MiSeq_SOP). Amplicon chimeras were examined using the UCHIME algorithm [12] implemented within mothur. Genus-level taxonomy was visualised using the R package ComplexHeatmap [13].

### FimH community profiling

Partial *fimH* gene sequences from urine samples were amplified and sequenced as previously described [14]. Chimeric sequences were removed using the UCHIME algorithm [12]. Clustering of *fimH* sequences was performed using default parameters (method = opti, cutoff = 0.03). Representative sequences from each cluster were used to identify the *fimH* type according to the online tool FimTyper (cge.cbs.dtu.dk/services/FimTyper/). Visualisation was performed using the R package ggplot2.

## Results

### Overview of the patient study group

Over a 2-year period, the clinical pattern of the UTIs of 39 women with refractory DO and a history of rUTI were closely observed. All patients gave MSU samples at the time of their LUTS exacerbation during this period. Nine patients had persistent recurrent bacteriuria and/or classical cystitis and were followed for the subsequent 24-month study period. These patients were postmenopausal and most used topical vaginal oestrogen cream (except for one woman with a history of previous breast cancer); median age was 75 years (interquartile range [IQR] 68.5–79.5 years; Table 1). The most frequent symptoms of UTI that patients volunteered were worsening of urgency, frequency and urge incontinence.

### Culture-based analysis of urinary samples

Close monitoring of the 9 patients revealed an exacerbation of LUTS symptoms or urge incontinence over the 24-month study period. A total of 102 samples were collected; 70 were confirmed UTIs (average of 8 UTIs per woman, median = 6, IQR = 3–13.5, range 3–17), 27 were reported as mixed growth and 5 did not yield significant growth (Table 1). A timeline depicting the diagnosis and treatment of UTI incidents over the 15-month period from June 2014 to September 2015 is summarised in Fig. 1. Within the limitations of timing, patient availability and onset of symptoms, 38 urine samples were also collected for both routine microbiological diagnosis and culture-independent 16 s rRNA amplicon sequencing (Fig. 1). Microbiological culturing identified a single dominant organism in 17 (44.7%) of samples; *E. coli* (*n* = 11),* Enterococcus faecalis* (*n* = 2),* Klebsiella pneumoniae* (*n *= 2), and* Streptococcus* spp. (*n* = 2). A further 16 samples (42.1%) were reported as “mixed growth”; common organisms included *Morganella*, *Klebsiella*, *Staphylococcus* and *Enterococcus*. Five samples did not yield significant growth. Resistance to ≥1 type of antibiotic was seen in 7 out of 9 of the women (78%) and 14 out of 17 of the samples (82.4%). Three patients had documented changes in the infecting organism over time, whereas the remaining patients were infected with a single persistent organism.

### Culture-independent analysis of urinary samples

**Fig. 2:**
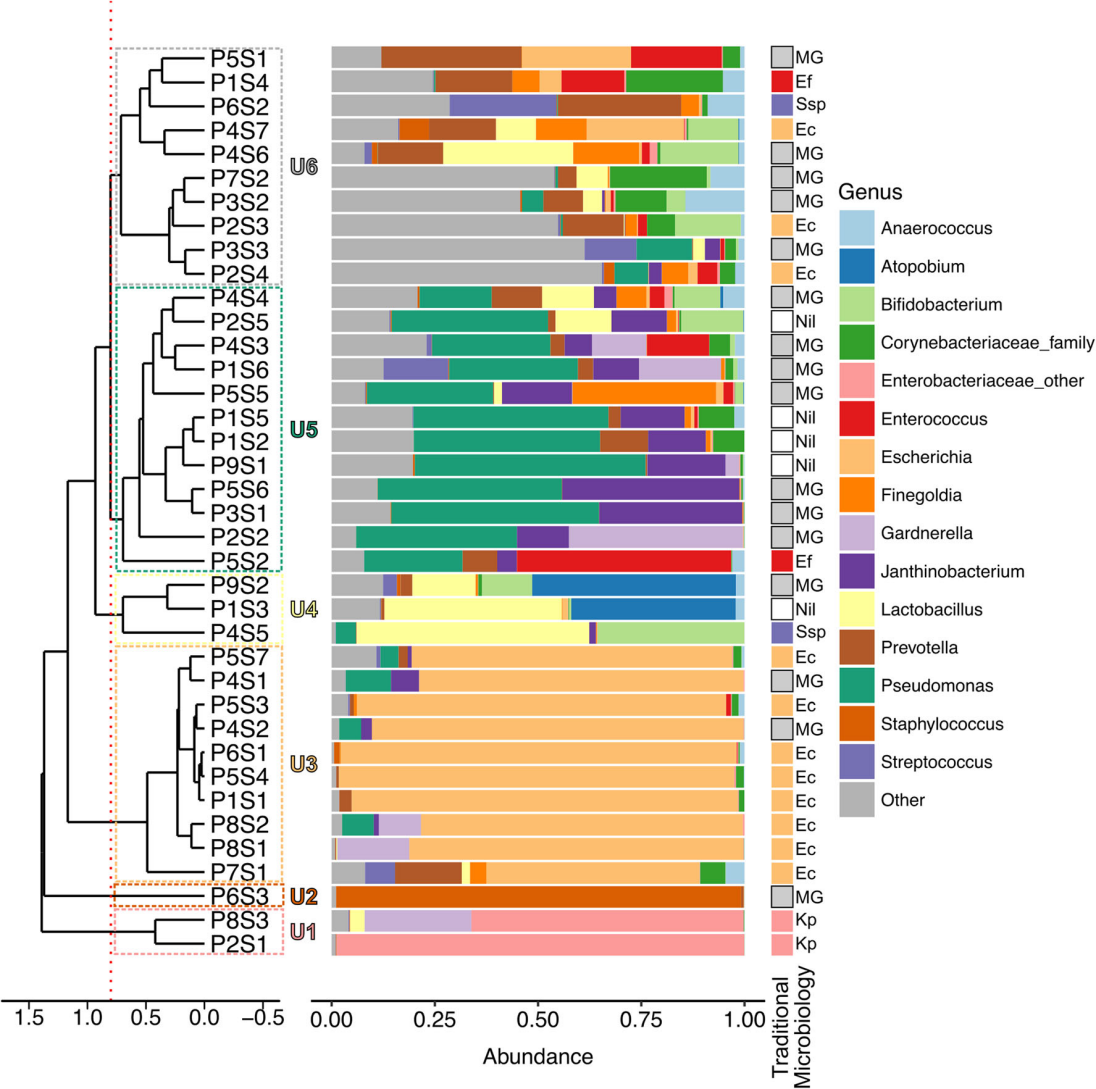
Clustering of urinary microbial profiles into six urotypes as demonstrated by the dendrogram (*left*) and by the dominant bacterial taxa present as depicted in the histogram (*middle*). Traditional microbiology results were also shown (*right*) to demonstrate the concordance between two different methods. The urine samples are identified by patient (*P*) number followed by sample (*S*) number from that patient. The dendrogram was based on the Euclidean distance between urine samples and the samples were clustered at ≥80% similarity, as shown by the* red dotted line* (six urotypes). The histogram showed the relative abundance of sequences coming from the most 15 abundant bacterial taxa identified across all samples. Bacteria were classified to genus level with the exceptions of* Enterobacteriaceae_other* and* Corynebacteriaceae_family*, which contain bacteria that could only be classified to family level. The* Other* category includes the remaining bacterial taxa, including unclassified sequences

The diversity of the urinary microbiota in the 9 patients was investigated by examining the 38 urine samples using 16S rRNA amplicon sequencing. A median of 25 genera of bacteria (IQR 21–36) per patient was detected. Based on the relative abundance of the top 15 bacterial taxa, the samples clustered into six different urotypes (samples in the same urotype shared ≥80% similarity by Euclidean distance; Fig. 2). The most common urotype was U5 (*n* = 12; dominated by Corynebacteriaceae), followed by U3 (*n* = 10; dominated by *Escherichia*), U6 (*n* = 10; diverse organisms), U4 (*n* = 3; dominated by *Lactobacillus*), U1 (*n* = 2; dominated by *Klebsiella*), and U2 (*n* = 1; dominated by *Staphylococcus*). All patients had at least two and up to four different urotypes, suggesting a continuous flux in their urinary microbiota (Fig. 1). Overall, the most abundant organism identified by culture-independent analysis showed high concordance with the dominant organism identified by traditional microbiology culturing.

### *E. coli* strain level identification reveals different patterns of rUTI

**Fig. 3:**
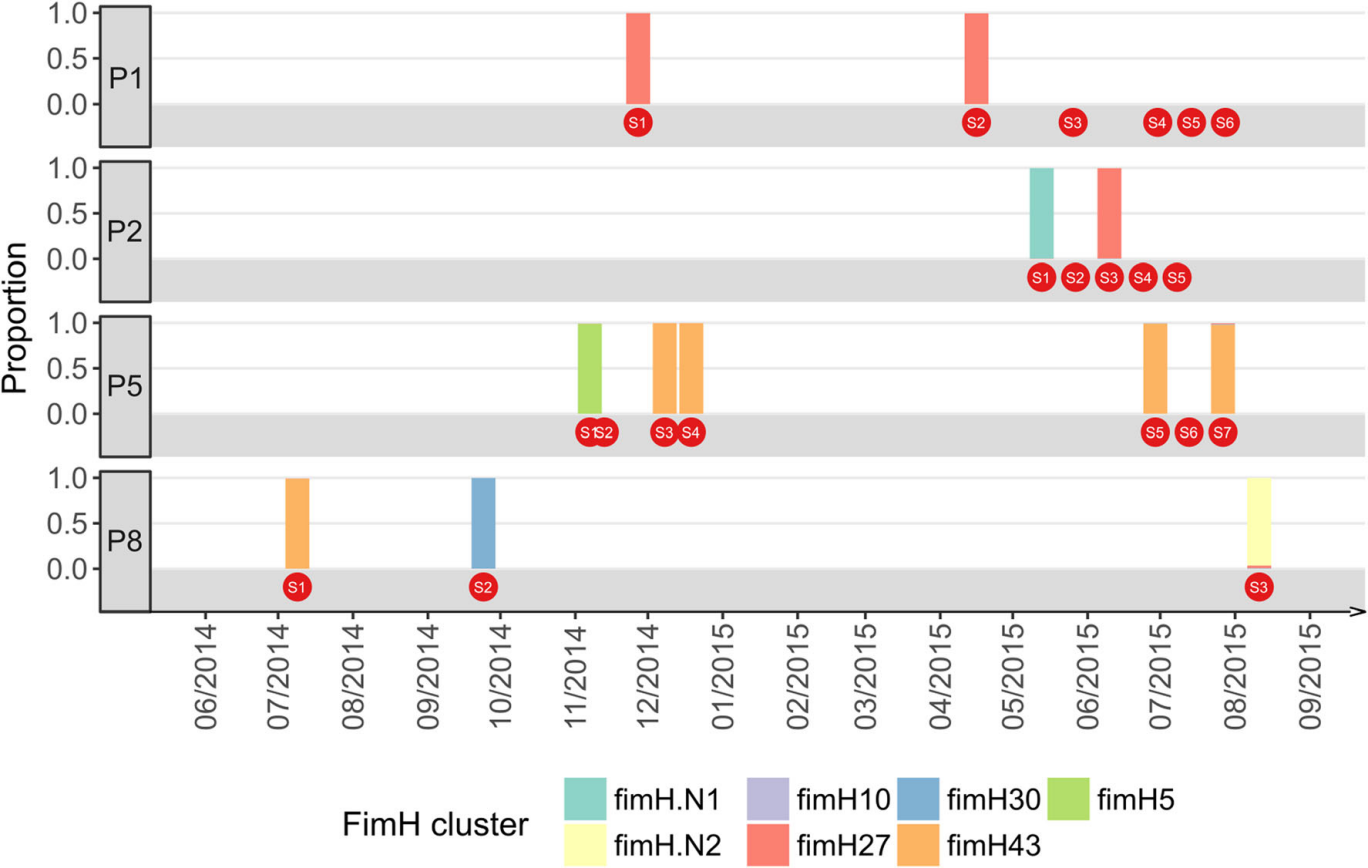
The dominant *fimH* alleles detected in samples from each patient. Only patients with 2 or more positive *fimH* PCR samples are shown. FimH alleles were identified by sequence similarity search against the *fimH* database provided by FimTyper; two alleles fimH.N1 and fimH.N2 were not found in this database

*Escherichia* was the most common genus detected in our patient cohort, and was identified in multiple samples from 4 patients based on 16S rRNA community profiling (patients 1, 2, 5 and 8). We further examined these *Escherichia*-positive samples using the *fimH* gene as a strain-level marker to distinguish between an rUTI with the same strain and a new infection. Overall, our data revealed examples of both of types of UTI (Fig. 3). Patient 2 (two different strains identified over a 1-month period) and patient 8 (three different strains identified over 12 months) represent examples of patients with urge incontinence suffering rUTI with new strains. In contrast, patient 1 (identical strains identified >4 months apart) and patient 5 (identical strain identified four times over an 8-month period) provide examples of patients with urge incontinence suffering from rUTI with the same strain. This finding in these two patients is suggestive of a reservoir for the persistence of a single infecting strain.

## Discussion

Women with severe refractory urge incontinence suffer an overwhelming burden of rUTI. Here, we have documented the progression of UTI in a cohort of such patients and correlated this with changes in their urinary microbiota.

Increasing evidence suggests that the urinary microbiota might play a role in the pathology of urge incontinence and refractory DO [3–5, 8]. Traditionally, clinicians in the field of urogynaecology have not recognised that women without the classical symptoms of dysuria and foul-smelling urine may nevertheless have recurrent, and often multi-resistant, bacterial cystitis. Our findings demonstrate that UTI is common in these patients, and highlight scenarios for rUTI with either the same persistent strain or a new infecting strain. Instances where refractory DO patients suffer from rUTI with the same strain suggest the existence of a chronic reservoir, with the source (i.e. bladder, urethra, vagina or bowl flora) being a contentious topic. In experimental mice, *E. coli* can replicate in superficial bladder epithelial cells as intracellular bacterial communities (IBCs) and survive intracellularly in a quiescent non-replicating state [15]. Evidence for the existence of IBCs and quiescent bacteria has been reported in human patients [8, 16], supporting the possibility that the bladder could serve as a reservoir for rUTI.

Our study specifically aimed to investigate women with refractory DO. Pearce et al. [4] previously demonstrated that there is a marked difference in the diversity of the microbiome of women with urge incontinence compared with healthy women with no proven history of UTI, thus negating the need for us to include a comparative control group in our study. Overall, our results demonstrate that refractory DO patients do have a urinary microbiota and we hypothesise that the composition of this microflora might have an impact on the incidence of rUTI and their lack of response following treatment with antimuscarinic drugs. It remains to be determined whether certain urotypes, as characterised in this work and by others [4], are associated with worsening patient outcomes. We also note that ~40% of the MSU specimens examined by routine pathology were reported as “mixed growth”, suggesting that the focus on reporting a single dominant organism might be open to question in patients with refractory DO who suffer from rUTI.

One limitation of this study was the use of MSU instead of catheter specimen urine (CSU), which may have contributed to an increased diversity in the urinary microbiome owing to possible contamination. However, careful labial toilet was employed, and catheterisation of patients who were at a high risk of bacterial infection was not ethically feasible as per our local ethics committee. Analysis of non-UTI CSU samples in a separate study [17] demonstrated that the urinary diversity of patients with urge incontinence who do not respond to VESIcare is eight times greater than normal controls. Thus, it is reasonable to assume that MSUs collected in our study are not a gross overestimation of urinary diversity. Another limitation of this study was the pragmatic approach to urine collection and treatment because of the geographical constraints of our cohort, who often travel >2-h for a clinic visit. Thus, non-discriminatory use of antibiotics was often initiated by general practitioners, resulting in variable antibiotic use (Fig. 1).

In summary, this study defines the existence and composition of the urinary microbiota in patients with refractory DO. We show that many of these patients suffer from frequent episodes of rUTI, suggesting that persistent bladder colonisation might contribute to the pathology of their chronic condition.
